# Multiple Variant Calling Pipelines in Wheat Whole Exome Sequencing

**DOI:** 10.3390/ijms221910400

**Published:** 2021-09-27

**Authors:** H. Busra Cagirici, Bala Ani Akpinar, Taner Z. Sen, Hikmet Budak

**Affiliations:** 1 Crop Improvement and Genetics Research Unit, Western Regional Research Center, U.S. Department of Agriculture—Agricultural Research Service, Albany, CA 94710, USA; busra.cagirici@usda.gov (H.B.C.); taner.sen@usda.gov (T.Z.S.); 2 Department of Genomics and Genome Editing, Montana BioAgriculture Inc., Missoula, MT 59802, USA; aniakpinar@gmail.com

**Keywords:** wheat, SNPs, WES, variants, BCFtools, STAR, Bowtie2, BWA

## Abstract

The highly challenging hexaploid wheat (*Triticum aestivum*) genome is becoming ever more accessible due to the continued development of multiple reference genomes, a factor which aids in the plight to better understand variation in important traits. Although the process of variant calling is relatively straightforward, selection of the best combination of the computational tools for read alignment and variant calling stages of the analysis and efficient filtering of the false variant calls are not always easy tasks. Previous studies have analyzed the impact of methods on the quality metrics in diploid organisms. Given that variant identification in wheat largely relies on accurate mining of exome data, there is a critical need to better understand how different methods affect the analysis of whole exome sequencing (WES) data in polyploid species. This study aims to address this by performing whole exome sequencing of 48 wheat cultivars and assessing the performance of various variant calling pipelines at their suggested settings. The results show that all the pipelines require filtering to eliminate false-positive calls. The high consensus among the reference SNPs called by the best-performing pipelines suggests that filtering provides accurate and reproducible results. This study also provides detailed comparisons for high sensitivity and precision at individual and population levels for the raw and filtered SNP calls.

## 1. Introduction

Advances in next-generation sequencing technologies have paved the way for improved genomic studies, providing enormous amounts of high-quality data in a fast and affordable manner [1]. Whole exome sequencing (WES) is one such advance, which focuses on capturing only exonic regions of the genome [2], and since its development, has been widely used to identify and understand structural variations of many disease-causing mutations [3,4]. Many molecular markers have been developed to highlight variation linked to characterized eco-, agronomically important traits, showcasing the importance of innovating technology for gene variant capture [5]. A benefit to this technology is that it only captures ~1–2% of the whole genome depending on species [6], focusing solely on regions that code for protein sequences, further increasing cost-effectiveness [7,8]. As a result of its economic viability and availability of high-quality data, WES is rapidly becoming a standard approach for detecting gene variants, where the major challenge of accurate and reproducible variant detection has shifted toward improving computational pipelines.

The process of variant discovery is composed of two major steps: Read mapping and variant calling, in which the aligner maps the reads to a reference genome and the caller determines the variant position and assigns a genotype [9]. Over the years, several new bioinformatic pipelines and computational tools have become available, enabling better analysis and interpretation of WES data. The most widely used aligners include Burrows–Wheeler transform (BWT)-based aligners such as BWA [10,11], Bowtie2 [12], and Hisat2 [13] as well as hash table based aligners such as GSNAP [14] and Novoalign. The most widely used variant callers include SAMtools/BCFtools [15] and VarScan2 [16]. However, different combinations of aligners and variant callers have demonstrated conflicting results for the superiority of one tool over another [17].

The performance of different aligners and variant callers have previously been assessed [18,19,20] to better characterize their potential in different organisms including humans, where exome sequencing plays a large role in clinical settings [2]. Comparative analysis of the variant calling pipelines in plants is limited to diploid species including tomato and *Arabidopsis thaliana* [21,22]. However, this is yet to be assessed in polyploid species. Even though similar WES computational pipelines have been used in both human and plant genomics to date, structural differences in the polyploid genomes impose new challenges on this. Aside from being highly repetitive, polyploid plant genomes possess nearly identical subgenomes and hence are more complex to analyze. Allohexaploid *Triticum aestivum* (wheat) is among the most important crops worldwide. Wheat has a large and complex genome [8] where its complexity partially arises from its highly repetitive homeologous chromosomes [23]. The hexaploid wheat is composed of three subgenomes with a high degree of collinearity and sequence conservation for the expressed genes [24]. Considering the conservation of the expressed gene sequences among the homeologous loci, reads mapping to multiple loci would be expected for exome sequencing data. As such, the performance of variant callers in detecting true variants in combination with the aligners remains to be evaluated in polyploid species.

With the recent release and availability of reference genomes [8], the wheat genome has become more accessible for the characterization of structural elements with possible regulatory functions. Although genomic resources have recently become richer [1,25,26], there is still no consensus regarding the optimal tool combinations for read alignment or variant calling. Given the current reference genome annotations and the availability of several advanced bioinformatic tools, the question has now become a matter of choice: Which tools to use? Providing an answer is not an easy task, especially when considering new users. As with every analysis, there is a need to ensure that results are both reliable and reproducible. This highlights a need to evaluate the bioinformatic pipelines for WES variant calling in wheat. Hence, this study aims to meet these needs by assessing the overall performance of various computational methods in a whole exome sequencing (WES) analysis pipeline using wheat exome data. 

## 2. Results

### 2.1. Datasets and Pipelines Evaluated

**Figure 1:**
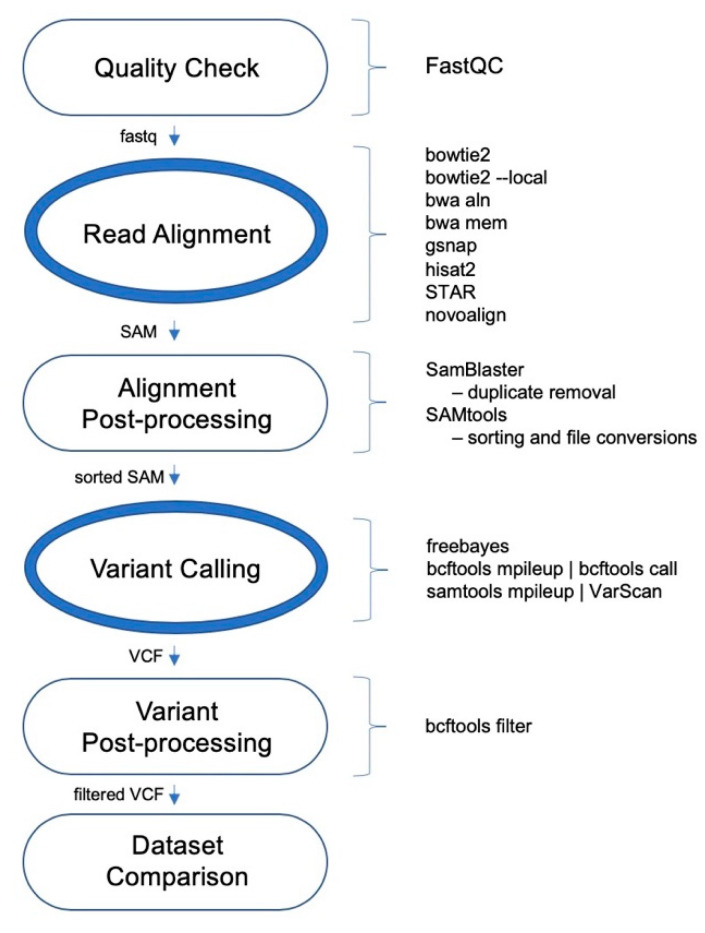
Schematic of the variant calling pipeline used.

The performances of 24 variant calling pipelines were evaluated using whole exome sequencing (WES) data for the wheat HAPMAP data [25], WhealBI dataset [26], where 1000 wheat exomes including both landraces and elite cultivars were sequenced [1] and 48 elite wheat cultivars. The schematic of general variant calling pipeline is shown in Figure 1. The number of read pairs ranged between 55.1 M and 77.5 M, totaling 3.05 B, whereas the number of unique read pairs ranged between 26 M and 35.9 M, totaling 1.51 B. All t48 WES datasets were aligned to the IWGSC wheat reference genome v1.0 separately using a range of aligners outlined below.

The aligners include both an old and new version of BWA; bwa aln and bwa mem, which from this point on will be referred to as BWA-backtrack and BWA-mem, respectively. Both functions of Bowtie2, Bowtie2 and Bowtie2-local were also included. Other aligners included Hisat2, GSNAP, STAR, and Novoalign. For variant calling, the three tools included were FreeBayes, VarScan, and BCFtools. Using different combinations of the eight aligners and three variant callers (Figure 1), a total of 24 pipelines was assessed.

### 2.2. Aligners Provided High Percentage of Paired-End Alignments with Varying Computational Cost 

**Figure 2:**
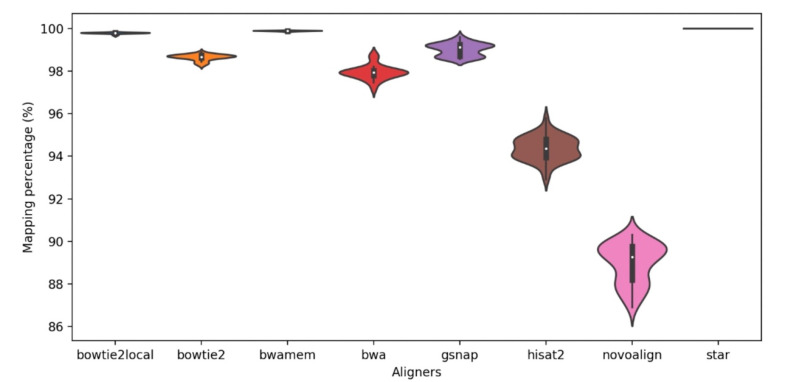
Mapping percentage of the eight aligners; Bowtie2-local (bowtie2local), Bowtie2, BWA-mem (bwamem), BWA-backtrack (bwa), GSNAP, Hisat2, Novoalign, and STAR.

The eight read aligners were compared at their suggested settings by their basic mapping statistics such as mapping percentages, coverage, and computation time. Overall, all aligners provided high mapping percentages with an average of >89% paired-end mapping and with a standard deviation up to 0.93 (Figure 2). Among all the aligners, STAR exhibited the highest percentage of paired-end alignments (100%) to the reference genome in all 48 samples. For Bowtie2-local and BWA-mem, >99% of the read pairs mapped to the reference genome for all 48 samples. The lowest alignment percentage (86.9%) was observed in one sample alignment using Novoalign.

The average run time of the aligners was compared across all samples. Table 1 shows the aligners and the average run times per exome data. Observed run times varied greatly from minutes to weeks. Due of the potential of longer calculations, longer run times were expected for aligners with high mapping percentages and vice versa; however, the results showed the opposite, with optimality in mapping algorithms. Interestingly, STAR showed the highest mapping percentage as well as the shortest computation time. Novoalign, on the other hand, showed the lowest mapping percentages and the longest run times. 

### 2.3. Number of Variations Varied Greatly among the 24 Pipelines

All three variant callers were run in combination with each of the eight aligners. Each variant caller was run according to their recommended settings using built-in functions and standard filtering if applicable (Methods S1). The number of SNPs called varied greatly among the variant calling pipelines (Table S1). Note that read aligners and variant callers in a pipeline are separated with an underscore symbol. The highest and the lowest number of SNPs were called by the GSNAP_BCFtools (5,079,109) and Hisat2_VarScan (143,629) pipelines, respectively. The number of SNPs called across all 48 samples ranged from 3,834,097 to 7,135,397 for GSNAP_BCFtools, and from 88,545 to 195,879 for the Hisat2_VarScan pipeline. 

**Figure 3:**
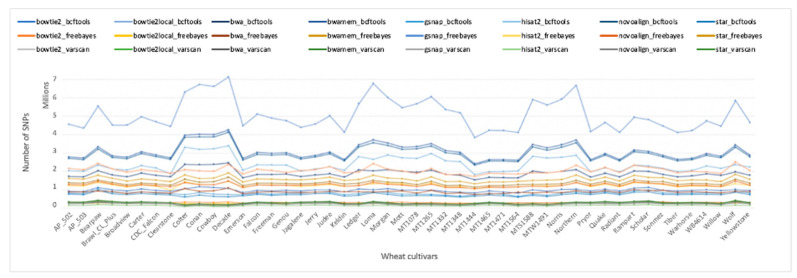
Number of SNPs called by 24 pipelines for 48 elite wheat cultivars. BCFtools pipelines were colored in blue, FreeBayes pipelines yellow/red, and VarScan pipelines in green/gray.

As shown in Table S1, different aligner and variant calling tool combinations can have a profound influence on the final set of variants. In fact, the results demonstrated that different variant callers identified different sets of variants from the same input files (Table S1). Therefore, the choice of the variant caller is a crucial step in selecting the optimum variant calling pipeline. Indeed, the initial screenings suggest that the number of SNPs called is more dependent on the variant caller rather than the read aligner. Figure 3 shows the number of SNPs called by each pipeline for all 48 samples. In general, the BCFtools pipelines called the highest number of SNPs across all aligners except STAR, which returned the highest number of variations when in combination with FreeBayes (Table S1). VarScan pipelines, on the other hand, were evidently the most conservative pipelines and called the lowest number of SNPs on average in combination with all aligners, except that of Bowtie2-local. The difference in the total number of SNPs called might be associated with the differences in the recommended settings of each variant caller such as the stringent filtering parameters of VarScan, as opposed to the less stringent parameters of FreeBayes and BCFtools (Methods S1). However, stringent filtering parameters alone do not explain the higher number of variations called by STAR_FreeBayes than the STAR_BCFtools pipeline (Table S1). Although FreeBayes standard filtering is more stringent than BCFtools, FreeBayes called more SNPs (1,581,001) than BCFtools (733,697) for the STAR alignments. Similarly, although VarScan applies a more stringent filtering than FreeBayes, FreeBayes called fewer SNPs than VarScan for Bowtie2-local alignments (Table S1), suggesting that the default stringent filtering of VarScan does not always result in the lowest number of variations called. A parameter fine tuning was applied to each pipeline later in this manuscript to adjust hard filtering parameters for all pipelines and compare the accuracy of the variant callers for high quality SNPs only.

### 2.4. Diversity among Pipelines

**Figure 4:**
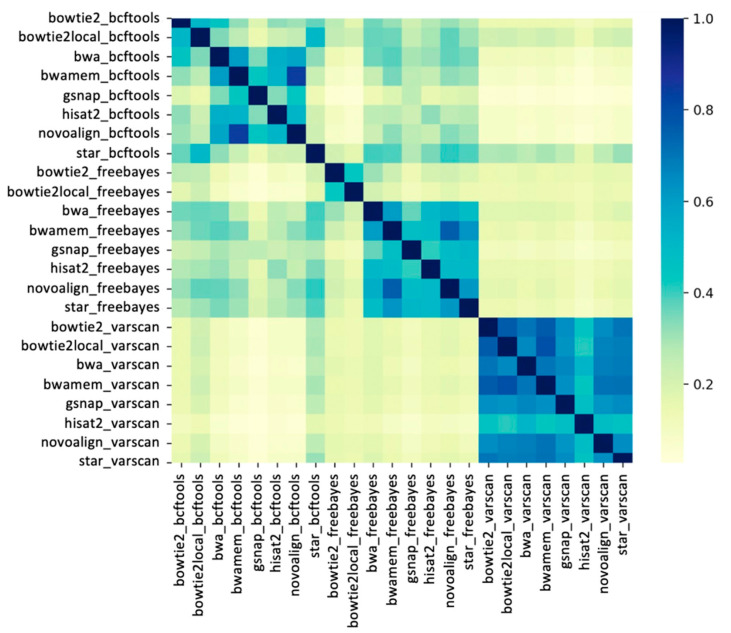
Heatmap showing the similarity between pipelines. The Jaccard indexes between pairs of pipelines were calculated for each sample and averaged across all 48 wheat samples. Color bar shows the Jaccard indexes between 0 and 1, where 1 indicates the identical pipelines.

The variant site concordance was assessed among the 24 pipelines using the Jaccard index metric for each of the 48 samples. For each sample, the Jaccard index of each aligner/caller pair was calculated based on the presence and absence of SNPs. The average Jaccard indexes across all 48 samples between each pipeline are shown in Figure 4. The results showed a clustering among variant callers, indicating that global similarities between variants called by different pipelines are largely dependent on variant callers. The highest Jaccard indexes (>0.7) were observed among most of the VarScan pipelines. However, VarScan pipelines are highly dissimilar from the pipelines that use FreeBayes and BCFtools, except in the following cases: STAR_BCFtools and Bowtie2-local_BCFtools. Interestingly, the STAR_BCFtools and Bowtie2-local_BCFtools pipelines showed higher similarity to the FreeBayes and VarScan pipelines than the other BCFtools pipelines.

Interestingly, the aligners BWA-mem and Novoalign showed the highest similarity in combination with any of the three variant callers (Jaccard index >0.7). Although BWA-mem and BWA-backtrack are different functions of the same aligner, BWA-mem showed the highest similarity to Novoalign. Additionally, relatively high Jaccard indexes (>0.5) were observed between different functions of the same aligners; for example, between BWA-backtrack and BWA-mem pipelines, and between Bowtie2 and Bowtie2-local pipelines.

### 2.5. Hard Filtering Eliminated Most of the False Positive Predictions

The false positive prediction performance of the variant calling pipelines was assessed using the exome sequencing data of one of the cultivars, Sonmez, against the preliminary sequence scaffolds of the same cultivar (unpublished data). Any variants detected are likely to be artifacts since both the genome and the exome sequencing data derive from the same cultivar. Initial results showed that regardless of the pipeline, variant calling is highly error-prone and resulted in a number of false positives (FP) in each of the pipelines even within the same genetic material (Table S2). The number of FP SNP calls varied greatly among the variant calling pipelines before filtering. Similar to the observation with the wheat genome reference sequence, the GSNAP_BCFtools pipeline called the highest number of SNPs (3,662,264) for the Sonmez genome and the Hisat2_VarScan pipeline called the lowest number (52,101) of SNPs (Table S2). Although the number of SNPs called within the same genetic material was less than the number of SNPs called from the wheat genome, the results here suggest that the standard filtering parameters included in the variant callers are not sufficient to eliminate false-positive calls and all variant calling pipelines require further variant filtering to eliminate the false positive predictions.

Variant filtering is one of the key steps in variant calling, which minimizes the number of false positive as well as the true-positives. Many variant callers have built-in variant filtering parameters as the default. However, these variant callers aim to report as many true variant calls as possible without losing too much sensitivity. Repetitive and paralogous sequences like allohexaploid wheat genome can give rise to high numbers of false positives. The filtering parameters were optimized to eliminate the filtering effect of the different variant callers and to eliminate most of the false-positive calls. The first filtering parameter was the strand bias [27], where the variants were removed if more than 90% of the alternative reads mapped to one strand. Remaining variants were filtered by genotype quality (<20) and by depth (<10). Additional filtering on higher depth values or on different parameters like mapping quality did not completely eliminate the false-positive calls. Therefore, the filtering parameters were empirically adjusted to minimize the detection of false-positive calls while preserving the sensitivity for the true-positives. 

On average 91%, 86%, and 28% of the false positive SNPs were filtered in the BCFtools, FreeBayes, and VarScan pipelines, respectively (Table S2). After filtering, the average number of false positive SNP calls was similar among the BCFtools (102,322), VarScan (95,763), and FreeBayes (93,929) pipelines. FreeBayes showed the highest variation across the aligners. The average number of false positives was the highest for the GSNAP_FreeBayes pipeline (194,601) and was the lowest for the Bowtie2_FreeBayes pipeline (12,083) among the 24 variant calling pipelines. Since VarScan already applies stringent filtering as a default, these filtering parameters reduced the false positive predictions at their lowest in the VarScan pipelines. Overall, these filtering cutoffs eliminated the vast majority of the false-positive calls and resulted in a similar average number of false positives among the variant callers. 

### 2.6. Construction of the Reference Dataset

Ideally, a gold standard dataset with all SNP positions known should be used for comparison purposes; however, such a dataset is not yet available for wheat. Therefore, a list of reference SNP positions was compiled from four independent wheat variation datasets to evaluate the prediction accuracy of variant calling pipelines. There are several SNP datasets available, each including tens to thousands of wheat samples. Variant calling pipelines and sample varieties differ greatly among these studies. For example, the wheat HAPMAP data were constructed from 62 samples using Bowtie2 and BWA-backtrack as an aligner [25], whereas the WhealBI dataset was constructed from 487 samples using BWA-mem as an aligner [26]. In another study, 1000 wheat exomes including both landraces and elite cultivars were sequenced [1], leading to a highly comprehensive SNP dataset. 

**Figure 5:**
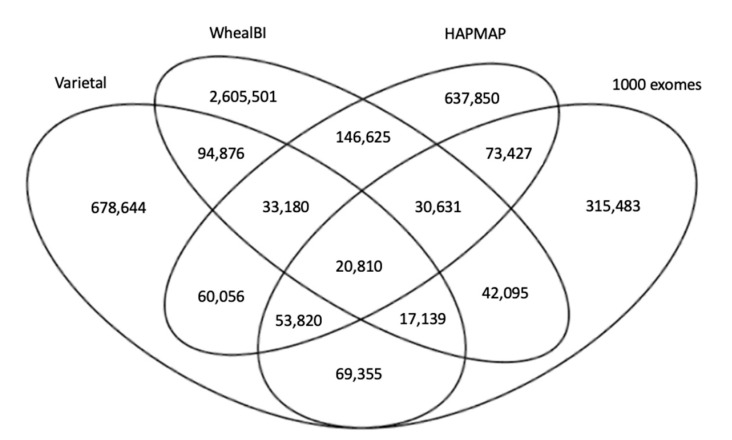
Venn diagram representing the consensus among four large-scale variation studies in wheat.

Ongoing efforts to unravel important elements of the wheat genome have revealed vast numbers of variants; however, little consensus among data of different consortiums points to the lack of a comprehensive SNP dataset for wheat. Figure 5 shows the number of common and specific SNP positions in the four large scale SNP datasets used in this study. More than 4.8 M (4,879,492) SNP positions were presented, where only 20,810 (0.43%) were common among all four datasets; 134,574 (2.76%) were common among three datasets; and 485,171 (9.97%) were common in at least two datasets. For each of these datasets, genotyping error rates were estimated to be smaller than ~2% by the authors [1,25,26] as validation of the SNPs called. As these studies include different varieties, every SNP position in four publicly available datasets was retrieved to increase variation coverage. The variety of samples and variant calling pipelines in these independent studies provided robustness for this study. 

### 2.7. Assessment of the True-Positive Prediction Performance of the Pipelines at the Sample Level

The SNPs identified from the 48 elite wheat WES samples were subjected to variant filtering for further analyses. The average number of SNPs called from the 48 WES data varied greatly among variant calling pipelines even after filtering (Table S1). On average, 89%, 87%, and 31% of the SNPs were filtered from the BCFtools, FreeBayes, and VarScan pipelines, respectively. The highest and the lowest numbers of SNPs were called by the GSNAP_BCFtools and Bowtie2-local_FreeBayes pipelines, respectively. 

To infer the true-positive prediction performance of the pipelines at the sample level, the SNP positions identified in each sample were compared to the reference dataset. The results showed that SNP filtering eliminated 71%, 74%, and 30% of the reference SNP calls on average from the BCFtools, FreeBayes, and VarScan pipelines, respectively. On the other hand, 95%, 92%, and 63% of the nonreference SNP calls were filtered. These results suggest that SNP filtering eliminated most of the false SNP calls; however, at the cost of losing true-positive calls. 

The average number of TP SNPs per sample was the highest in the BCFtools pipelines (118,265 following BWA-mem and 107,157 following GSNAP) and the lowest in the FreeBayes pipelines (17,524 following Bowtie2-local and 25,580 following Bowtie2) (Table S3). In general, the number of TP SNP calls were higher in the pipelines that called a higher number of SNPs (both true-positives and false positives). 

In order to assess the overall prediction performance of the pipelines at the sample level, the precision, sensitivity, and F1 score were calculated for each of the 48 WES data. Average accuracy metrics are shown in Table S3. The results showed that the BWA-mem_BCFtools pipeline provided the highest sensitivity and F1 score for each of the 48 WES data. However, its precision (~0.47) stayed lower from the average precision (0.53) among the 24 pipelines. The highest precision (~0.73) was obtained from the Bowtie2_FreeBayes pipeline for all of the 48 WES data; however, the total number of TP SNPs, sensitivity, and the F1 score per sample were way below the average scores among the variant calling pipelines. Finally, the pipelines were selected for general use that provided above the average scores per sample for (i) precision, (ii) sensitivity, and (iii) F1 score. These pipelines, sorted by the total number of TPs, were STAR_BCFtools, BWA-backtrack_BCFtools, Bowtie2-local_BCFtools, Bowtie2_BCFtools, and BWA-backtrack_varscan. 

### 2.8. Population Level Comparison of the Variant Calling Pipelines

Other than in the individual datasets, the performance of the variant calling pipelines were assessed at the population level using all the wheat samples. SNP calls from 48 WES data were merged for each of the 24 variant calling pipelines using the BCFtools merge function. Merging SNPs from the individual samples for each variant calling pipeline resulted in a more comprehensive set of SNPs. Total number of SNP positions called by the pipelines was increased by 6- to 10-fold from the average number of SNPs per sample, indicating that most of the SNP positions were called from more than one sample. The ranking of the pipelines based on the accuracy metrics were the same as the ranking at the sample level (Table S3). 

The results showed that there is usually a trade-off between precision and sensitivity. The highest precision was observed in the Bowtie2_FreeBayes pipeline with a cost of low precision and F1 score. The highest number of TP calls, sensitivity, and F1 score were observed in the BWA-mem_BCFtools pipeline with a cost of low precision. The pipelines with the highest precision may be better suited for the identification of a short list of high confidence variations rather than novel variations and/or complete sets of variations for a GWAS study. Given the highest F1 score, sensitivity, and the higher number of TP calls, the BWA-mem_BCFtools pipeline might be better suited for obtaining a comprehensive set of SNPs, allowing the false calls to some extent for a GWAS study. For the optimum results, this study suggests the five pipelines providing above average scores for each of the accuracy metrics. These pipelines were ranked by the highest number of TP calls as follows: STAR_BCFtools, BWA-backtrack_BCFtools, Bowtie2-local_BCFtools, Bowtie2_BCFtools, and BWA-backtrack_VarScan. 

### 2.9. Final Comparison of the Five Best-Performing Pipelines

**Figure 6:**
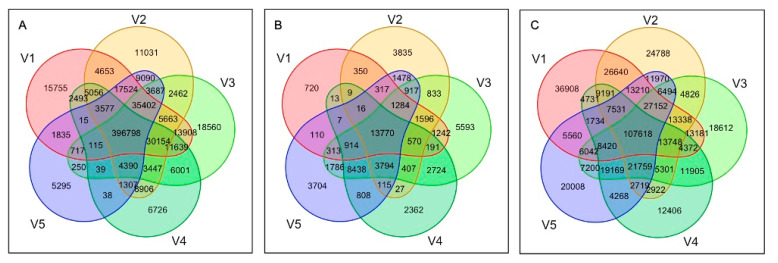
Venn diagram depicting the number of SNPs identified as true-positives from the best performing pipelines. Number of overlapping SNPs with reference set were shown for (**A**) variants merged for 48 samples, and (**B**) variants merged for 48 samples and filtered by missing genotypes (**C**) variants merged for 48 samples and filtered by missing genotypes after imputation. V1: STAR_BCFtools, V2: BWA-backtrack_BCFtools, V3: Bowtie2-local_BCFtools, V4: Bowtie2_BCFtools, and V5: BWA-backtrack_BCFtools.

The best-performing pipelines were selected and the consensus among the true-positive variants was assessed at the population level. The best-performing pipelines included the BCFtools in combination with STAR, BWA-backtrack, Bowtie2-local, Bowtie2, and VarScan in combination with BWA-backtrack. Figure 6A shows the number of truly detected SNPs by these top five performing tools across all samples. Among the variants identified as true-positives, ~63% of the observations were common across the best-performing pipelines (Figure 6A) and only ~9% were uniquely identified by one of these pipelines, suggesting a consistency in the predicted variations by the top performing pipelines.

The variant sites called from >25% of the samples and with minor allele frequency (MAF) >0.01 were further investigated. Figure 6B shows the number of truly detected SNPs by these top five performing tools after variant site filtering. Total number of common SNPs among the top performing pipelines decreased to 24% after filtering and the number of unique SNPs increased to 28%. These results suggested that this additional filtering by missing genotypes and MAF did not improve consistency among the pipelines. Additionally, this filtering lowered the prediction accuracy metrics in all five pipelines. 

Recently, WES studies have employed imputation to resolve missing genotype calls based on common variants in a population. To prevent filtering true variant sites based on missing genotype calls, the missing genotypes in each of the top performing pipelines were recovered using a standard imputation by Beagle 5.1. Each variant calling pipeline was subject to imputation, and high confidence variant calls were retrieved by filtering variant positions with >75% missing genotypes and <0.01 MAF. Figure 6C shows the number of SNPs identified as true-positives among the high confidence set of imputed variants. After imputation, the total number of observations increased drastically (~9-fold). However, the percentage of common (23%) and unique (24%) SNPs across all observations in the best-performing pipelines did not show any improvement (Figure 6B), thus suggesting that the consistency among the pipelines cannot be improved by imputation alone. On the other hand, imputation alone increased the number of TP calls identified by the pipelines (Figure 6B,C), which consequently increased the sensitivity.

## 3. Discussion

The two main stages of variant discovery are read alignment and variant calling. There are a vast number of tools available for each stage. The present study compared 24 variant calling pipelines based on a combination of eight aligners and three variant callers. This study followed the manuals and the tutorials of each of the pipelines for the optimized parameters set by the developers. Additionally, the impacts of missing data imputation and variant filtering were evaluated. This comparative analysis aims to provide guidance as to how to choose the best variant calling pipeline for an individual dataset or multiple datasets.

Wheat has a highly repetitive allohexaploid genome that contributes to the reads mapping to multiple loci. Multi mapping reads are assigned by lower mapping scores and even eliminated if mapped to too many loci by the aligners. Variant callers take these scores into account when considering a variant site. Efforts to call variants from uniquely mapped reads in tetraploid peanut have resulted in limited success as this conservative approach severely limited the total number of SNPs to only 1765 SNPs [28]. Additionally, considering the highly repetitive and nearly identical subgenomes of allohexaploid wheat, this study included multimapping reads and allowed the algorithms of variant callers to make supervised decisions based on the mapping scores. Among the variant callers, only FreeBayes offers the ploidy parameter; however, the results were highly variable in the FreeBayes pipelines when combined with different aligners (Table S1). The variation among the number of SNPs called by each pipeline suggests the lack of consensus among the pipelines and further suggests that many of the calls might be false-positives filtered by other pipelines, and thus further refinement of the variants is required for all the pipelines.

The false positive prediction rates of the 24 pipelines were assessed by using the genome and the exome sequencing data of the one of the wheat elite cultivars, Sonmez. The results showed that none of these pipelines were sufficient to eliminate false-positive calls in the raw data without filtering. Hard filtering by strand bias, depth, and quality greatly reduced the false-positive calls, resulting in a similar number of false-positives for each of the variant callers. Hard filtering resolved the discrepancy among the number of SNPs identified by each of the variant callers and eliminated the vast majority of the false-positives. It is important to note that hard filtering did not eliminate the false-positive calls completely and increasing stringency will also eliminate the true-positive calls. Therefore, this study assessed the prediction performance of the pipelines based on F1 score, precision, and recall. Later, the best performing pipelines were selected among the pipelines above the average scores for each of the metrics.

Different quality metrics might be preferred depending on the research question. High sensitivity is desirable when a high number of true-positive variations is required. BCFtools following the BWA-mem, GSNAP, STAR, and BWA and FreeBayes following GSNAP provided the highest sensitivity at the sample and at the population level analyses. However, many of these pipelines provided lower precision scores, which is required for a high quality of the variants without a background noise. FreeBayes following Bowtie2 and Bowtie2-local, VarScan following the Hisat2, and BCFtools following Bowtie2, Bowtie2-local, STAR, and BWA provided the highest precision. Considering both accuracy metrics as well as the harmonic mean of sensitivity and precision (F1 score), the best pipelines were determined for optimum use, providing higher precision and sensitivity compared to the remaining pipelines. The optimum results were obtained with the BCFtools following STAR, BWA, Bowtie2-local, and Bowtie2 and with the VarScan following BWA. Given the highest number of true-positives obtained and providing one of the highest scores for each evaluation metrics, the results of this study suggest STAR in combination with BCFtools and hard filtering for further studies. 

This study showed that the variant callers tested had a greater influence on the variation identification than the read aligners. Additionally, merging individual datasets increased the number of true-positive calls in the elite wheat population. The pipelines provided similar performances at the sample and population levels. The top performing pipelines provided consistent reference SNP calls where only ~9% of the SNP sites were uniquely identified by a pipeline. 

Additional filtering of the variant sites with >25% missing genotypes and <0.01 MAF resulted in an increased number of pipeline specific variants and provided lower precision and sensitivity. Imputation, on the other hand, recovered the lower scores and increased the number of TP calls. Therefore, this study suggests imputation prior to variant site filtering based on missing genotypes and allele frequencies. This study provided an assessment of 24 different variant calling pipelines based on the whole exome sequencing data of 48 elite wheat cultivars. The findings of this study serve the plant genomics studies for accurate variants that can be reproducible by many pipelines. 

## 4. Materials and Methods

### 4.1. Preparation of Wheat Exome Capture Libraries

Exome regions were captured with SeqCap EZ Developer Reagents (Roche Sequencing, Indianapolis, IN, USA). The libraries for 48 elite wheat cultivars were sequenced using a HiSeq 4000 Sequencing Kit version 1 (University of Illinois at Urbana Champaign, Roy J. Carver Biotechnology Center, Chicago, USA). Generated FASTQ files were demultiplexed with the bcl2fastq v2.17.1.14, which removes adaptors from the 3’-end of the reads. 

FastQC [29] quality assessment was successful for all of the metrics other than per base sequence content and sequence duplication. Per base sequence content failed at the end of reads, which is natural, as adaptor trimming introduces a composition bias at read ends. Fluctuations in the base sequence content for the first 10 to 15 bases were also generated by many sequencing platforms. Additionally, the sequence duplications levels were ~50% for most of the dataset, which corresponds to natural read duplication due to fragmentation bias for exome sequencing datasets [30]. 

### 4.2. Alignment Parameters

The overall pipeline starting from raw FASTQ files and ending with filtered VCF files is shown in Figure 1. After quality controls, WES reads for 48 wheat cultivars were aligned to the IWGSC Chinese Spring wheat reference genome assembly v1.0 separately using eight different aligners. Aligners included were Bowtie2 v2.3.4.1 [12], Bowtie2-local (Bowtie2-local), BWA aln/sampe v0.7.17 (BWA-backtrack) [11], BWA mem v0.7.17 (BWA-mem), GSNAP Version 2018-03-25 [31], Hisat2 v2.1.0 [32], STAR v2.6.1a [33], and Novoalign v3.09.00 (http://novocraft.com/, accessed on 15 September 2020). These aligners converted FASTQ files into raw SAM files. Multiple threads were used where available.

Alignments were processed further before variant calling to prepare a sorted and clean BAM file. First, PCR duplicates were marked from each SAM file using SAMblaster v0.1.24 [34]. Then, SAM files were converted into BAM files and sorted using SAMtools v1.9 [15]. Finally, group IDs were inserted according to file names using SAMtools. The detailed commands for each stage of these analysis are provided in Methods S1.

### 4.3. Variant Calling

Three variant calling methods were executed for each alignment (48 samples * 8 aligners): FreeBayes v1.2.0 [35], BCFtools call (BCFtools) v1.8, and VarScan v2.4.2 (SAMtools) [16]. The variant calling step was performed separately, as FreeBayes and VarScan required a very high memory usage for the merged files (>240 GB for 48 samples merged). Built-in functions and standard filtering parameters were applied to each tool as suggested by their tutorials (Methods S1).

Variant sites on an unknown chromosome were removed from further analyses using the BCFtools filter function. Single-nucleotide polymorphism (SNP) calls were extracted from the resulting VCF files using the BCFtools view function.

### 4.4. Filtering and Imputation

The BCFtools filter function was used to filter low quality SNPs based on alternate allele strand bias (>90%), quality (<20), and depth (<10) (Methods S1). A merged VCF file was prepared for each variant calling pipeline by merging individual VCF files of 48 wheat samples using the BCFtools merge function. 

For imputation, merged VCF files were subjected to Beagle software (v5.1) [36] with the effective population size parameter set to ‘ne = 1300’ according to previous estimates [37]. Additional filtering by <75% missing data and <0.01 minor allele frequency (MAF) was applied on the merged VCF files before and after imputation using the BCFtools filter function. 

### 4.5. High Confidence Reference Dataset

To create high coverage in the reference dataset, this study used SNP positions from four comprehensive SNP datasets: WhealBI variations [26], wheat HAPMAP data [25], 1000 wheat exomes project [1], and varietal SNPs identified by the Akhunov lab and the Dubcovsky lab, which can be obtained from the wheat-urgi database (https://wheat-urgi.versailles.inra.fr/Seq-Repository/Variations, accessed on 15 September 2020) and GrainGenes (https://wheat.pw.usda.gov, accessed on 15 September 2020) [38]. A reference SNP dataset was compiled from the SNP positions in these four datasets.

### 4.6. Evaluation Criteria

To assess performance of each variant calling pipelines, the accuracy metrics of sensitivity, precision, and F-score was used. True-positive (*TP*) calls were defined as reference variants called by the variant calling pipelines. False-positive (*FP*) calls were defined as variants called by the variant calling pipeline, which were not reference variants. False-negative (*FN*) calls were defined as reference variants that were not called by the variant calling pipeline. Accuracy metrics were calculated as follows:

$$precision=\frac{TP}{\left(TP+FP\right)}\;sensitivity=\frac{TP}{\left(TP+FN\right)}\;F1-score=\frac{\left(2*precision*recall\right)}{\left(precision+recall\right)}$$

In order to assess the similarity among pipelines, the Jaccard index between variant calling pipelines was calculated. The Jaccard index is also known as intersection over union and is calculated as the total number of variations at the intersection of the datasets divided by the size of the union of datasets. Jaccard indexes are distributed between 0 and 1, where 1 indicates identical datasets. Jaccard indexes were visualized on a heatmap using python.

## 5. Conclusions

Understanding genetic variations is vital for studying genetic and physical mapping, diversity, evolution, and breeding. However, as most variant calling tools have been developed and optimized to perform with diploids, additional steps need to be taken to streamline the process for plant species such as wheat, which has a complex polyploid genome. In this study, the 24 variant calling pipelines were evaluated and used at their suggested settings with newly sequenced whole exome data of 48 wheat cultivars. Considering that only a small overlap exists among the current comprehensive wheat datasets, the results of the present study will provide a better assessment of the variant calling pipelines for accurate and reproducible variant calls in polyploid species. These findings are intended to serve as more accurate variants that can be repeatable and reproducible by many pipelines.

## Figures and Tables

**Table 1:** Average run time of aligners per exome data.

Aligner	Average Run Time	Number of Threads
STAR	00–00:22:17	20
Hisat2	00–00:24:36	20
BWA-mem	00–01:10:26	20
Bowtie2	00–01:29:16	20
Bowtie2-local	00–01:39:30	20
BWA-backtrack	00–04:50:49	20
GSNAP	05–05:36:37	20
Novoalign	14–16:30:00	1

Run time: days-hours: minutes:seconds.

## Data Availability

The datasets are publicly available at URGI, and the International Wheat Genome Sequencing Consortium, and custom scripts and 48 wheat WES data are only available upon request from the corresponding author. Table S1 shows the number of SNPs called by each pipeline per sample. Table S2 shows the total number of SNP positions called from the 48 wheat WES data by the 24 pipelines. Table S3 shows the accuracy metrics of the performance of variant calling pipelines at both the sample and population levels. Method S1 contains the description of the variant calling pipelines.

## Supplementary Materials

The following are available online at https://www.mdpi.com/article/10.3390/ijms221910400/s1.
