# Supplementary material for: Multiple Variant Calling Pipelines in Wheat Whole Exome Sequencing
Source: Int J Mol Sci. 2021 Sep 27;22(19):10400. doi: 10.3390/ijms221910400 (PMC8509018; doi:10.3390/ijms221910400)
Supplement: Supplementary file 1 [file ijms-22-10400-s001.zip › Methods_S1.pdf]

Description of the variant calling pipelines

Specific command lines for each step of the analysis are provided below. Most tools were run at the default settings. For the remaining tools, we here provide detailed description of the parameters used. Note that, the defaults of the variant callers are different based on the optimization of their intrinsic algorithms.

Read Alignment

In order to align reads to the reference genome, reference genomes were indexed using index functions of each aligner. Multiple threads were applied where available. Below are commands for read alignment where the first line represents indexing of the reference genome and the following lines show the alignment:

STAR:

```
STAR --runMode genomeGenerate --genomeDir $starindex --genomeFastaFiles $genomefasta --
sjdbGTFfile $genomegtf --sjdbOverhang 99 --limitGenomeGenerateRAM 40000000000 --
runThreadN $threadN

STAR --genomeDir $starindex --readFilesIn $read1 $read2 --outFileNamePrefix
$filename.star.sam --runThreadN $threadN > $filename.star.sai
```

BWA (BWA-mem and BWA-backtrack):

```
bwa index $genomefasta

bwa mem -M -t 20 $genomefasta $read1 $read2 > $filename.bwamem.sam

bwa aln -t 20 $genomefasta $read1 > $filename.bwa1.sam
bwa aln -t 20 $genomefasta $read2 > $filename.bwa2.sam
samtools index $filename.bwa1.sam > $filename.bwa1.sai
samtools index $filename.bwa2.sam > $filename.bwa2.sai
bwa sampe -a 200 $genomefasta $filename.bwa1.sam $filename.bwa2.sam $read1 $read2 >
$filename.bwa.sam
```

Bowtie2 (Bowtie2 and Bowtie2-local):

```
bowtie2-build $genomefasta wheat_bowtie2_db

bowtie2 -x ../wheat_bowtie2_db -p 20 -1 $read1 -2 $read2 -S $filename.bowtie2.sam

bowtie2 --local -x ../wheat_bowtie2_db -p 20 -1 $read1 -2 $read2 -S
$filename.bowtie2local.sam
```

Hisat2:
hisat2-build $genomefasta wheat_hisat2_db

hisat2 -x ../wheat_hisat2_db -p 20 -1 $read1 -2 $read2 -S $filename.hisat2.sam

GSNAP:
gmap_build -d wheat_gsnap_db -D gsnapindex $genomefasta

gsnapl -A sam -d wheat_gsnap_db -D ../gsnapindex -t 20 $read1 $read2 > $filename.gsnap.sam

NovoAlign:
novoindex wheat_novoindex_db.ndx $genomefasta

novoalign -d wheat_novoindex_db.ndx -f $read1 $read2 -o SAM > $filename.novoalign.sam

Alignment Post-processing

Alignment files were further processed from SAM to BAM file formatting through duplicate marking, file conversion, and sorting. Command lines were as follows:

samblaster -M -i $filename.sam -o $filename.dup.sam
samtools view -bS $filename.dup.sam > $filename.dup.bam
samtools sort -m 120G $filename.dup.bam -o $filename.dup.sorted.bam
samtools addreplacerg -R $filename -o $filename"_RG.bam" $filename.dup.sorted.bam

Variant calling

FreeBayes: Following the manual (https://github.com/ekg/freebayes), we downloaded and ran FreeBayes at its suggested settings. In its tutorial, FreeBayes states that ploidy parameter should be set unless samples are diploid. Additionally, FreeBayes by default reports variations even with very low probability in the expectation that the results will be filtered. Instead of user-defined parameters, FreeBayes offers --standard-filters which is equivalent to --min-mapping-quality 30 and --min-base-quality 20. Command line was as follows:

freebayes -f $genomefasta --standard-filters --ploidy 6 $sortedbam > $sortedbam.freebayes.vcf

By defaults, indel realignment is on in FreeBayes. Other default parameters include the number of observations (-C 2), the percentage of the reads from a single sample (-F 0.2) and expected mutation rate (-theta 0.001) to filter variants.

VarScan2: VarScan calls variations from SAMtools mpileup files. To call SNPs using VarScan2, we first run SAMtools mpileup function and mpileup file was piped to VarScan to call SNPs using mpileup2snp function. Based on VarScan manual (http://varscan.sourceforge.net/support-faq.html), the default settings are optimized for exome data; therefore, we run VarScan at its default settings, except for –output-vcf 1 to receive output in vcf file format. Command line was as follows:

```
samtools mpileup -f $genomefasta $sortedbam | varscan mpileup2snp --output-vcf 1 >
$sortedbam.varscan.vcf
```

VarScan provides useful parameters, each set to defaults, for;

minimum read depth at a position (--min-coverage 8),
minimum supporting reads at a position (--min-reads2 2),
minimum base quality (--min-avg-qual 15),
minimum variant allele frequency (--min-var-freq 0.01),
minimum frequency to call homozygote (--min-freq-for-hom 0.75),
p-value threshold (--p-value 99e-02),
Ignore variants with >90% support on one strand (--strand-filter 1),

BCFtools: We performed variant calling using mpileup and call functions BCFftools. In its earlier tutorials (http://www.htslib.org/workflow/#mapping_to_variant), BCFtools suggested the use of BaseRecalibrator prior to variant calling. As the newest version of BCFtools include --redo-BAQ parameter to recalculate base alignment quality (BAQ), we used --redo-BAQ built-in function in BCFtools mpileup.

BCFtools call function was run at default settings with the options of -m to use the default calling method multiallelic caller -v outputs variant sites only -Ov output in uncompressed VCF file format -o $sortedbam.bcftools.vcf to define output file name. Command line was as follows:

```
bcftools mpileup --redo-BAQ -f $genomefasta $sortedbam | bcftools call -mv -Ov -o
$sortedbam.bcftools.vcf
```

Other default parameters include --max-depth 250 and --min-BQ 13.

Hard filtering

BCFtools filter function was run to filter variants with >90% allele bias, quality <20, and depth <10. The command line arguments for each of the pipeline were below:

From BCFtools pipelines:

```
bcftools filter -e 'QUAL<20 || DP<10 || DP4[2]/(DP4[2]+DP4[3])>0.9 || DP4[2]/(DP4[2]+DP4[3])<0.1 ' $f -o $f.filtered.DP10.QUAL20.strandbias.vcf
```

From VarScan pipelines:

```
bcftools filter -e 'GQ<20 || DP<10 || ADF/(ADF+ADR)>0.9 || ADF/(ADF+ADR)<0.1 ' $f -o $f.filtered.DP10.QUAL20.strandbias.vcf
```

From FreeBayes pipelines:

```
bcftools filter -e 'QUAL<20 || INFO/DP<10 || SAF/(SAF+SAR)>0.9 || SAF/(SAF+SAR)<0.1 ' $f -o $f.filtered.DP10.QUAL20.strandbias.vcf
```
