# Supplementary material for: Multiple Variant Calling Pipelines in Wheat Whole Exome Sequencing
Source: Int J Mol Sci. 2021 Sep 27;22(19):10400. doi: 10.3390/ijms221910400 (PMC8509018; doi:10.3390/ijms221910400)
Supplement: Supplementary file 1 [file ijms-22-10400-s001.zip › Table S2.pdf]

Table S2. Total number of SNP positions called from the 48 wheat WES data by the 24 pipelines.

	RAW			FILTERED			PERCENTAGE OF CHANGE		
	BCFtools	VarScan	FreeBayes	BCFtools	VarScan	FreeBayes	BCFtools	VarScan	FreeBayes
**BOWTIE2**	445,100	156,015	135,585	46,820	110,825	12,083	0.89	0.29	0.91
**BOWTIE2LOCAL**	587,062	178,703	121,019	68,952	126,846	15,222	0.88	0.29	0.87
**BWA**	1,078,528	112,222	431,928	94,653	81,880	53,624	0.91	0.27	0.88
**BWAMEM**	1,914,760	165,928	770,611	157,388	120,497	114,518	0.92	0.27	0.85
**GSNAP**	3,662,264	129,402	1,291,604	177,731	95,290	194,601	0.95	0.26	0.85
**HISAT2**	1,262,786	52,101	644,313	55,256	38,675	100,135	0.96	0.26	0.84
**NOVOALIGN**	1,914,005	121,513	819,156	135,406	85,319	118,316	0.93	0.30	0.86
**STAR**	455,970	147,185	934,211	82,368	106,774	142,930	0.82	0.27	0.85
