# Supplementary material for: Multiple Variant Calling Pipelines in Wheat Whole Exome Sequencing
Source: Int J Mol Sci. 2021 Sep 27;22(19):10400. doi: 10.3390/ijms221910400 (PMC8509018; doi:10.3390/ijms221910400)
Supplement: Supplementary file 1 [file ijms-22-10400-s001.zip › Table_S1.pdf]

**Table S1** Number of SNPs called by each pipeline per sample. Average, minimum, and maximum number of SNPs per sample called by each pipeline for 48 wheat cultivars (sample) are shown. RSD stands for relative standard deviation which is defined as the ratio of the standard deviation to the mean. Read aligners and variant callers in a pipeline are separated with an underscore symbol.

| | Before filtering | | | | After filtering | | | |
|---|---|---|---|---|---|---|---|---|
| | average | RSD | min | max | average | RSD | min | max |
| **Bowtie2_BCFtools** | 978,443 | 8 | 826,717 | 1,171,019 | 132,797 | 16 | 83,718 | 177,541 |
| **Bowtie2_FreeBayes** | 317,345 | 7 | 277,881 | 372,250 | 35,102 | 15 | 23,351 | 48,615 |
| **Bowtie2_VarScan** | 282,273 | 18 | 178,770 | 400,365 | 194,039 | 20 | 117,530 | 274,243 |
| **Bowtie2-local_BCFtools** | 814,822 | 10 | 662,044 | 1,053,343 | 156,294 | 17 | 99,565 | 212,912 |
| **Bowtie2-local_FreeBayes** | 248,147 | 8 | 213,940 | 291,484 | 28,000 | 15 | 19,159 | 37,958 |
| **Bowtie2-local_VarScan** | 306,528 | 19 | 192,098 | 436,306 | 209,776 | 20 | 126,537 | 298,749 |
| **BWA-backtrack_BCFtools** | 1,889,604 | 11 | 1,537,859 | 2,466,080 | 186,712 | 17 | 123,136 | 252,249 |
| **BWA-backtrack_FreeBayes** | 886,538 | 8 | 751,378 | 1,099,124 | 110,607 | 15 | 76,940 | 144,506 |
| **BWA-backtrack_VarScan** | 234,661 | 18 | 144,726 | 331,514 | 162,209 | 20 | 94,794 | 228,775 |
| **BWA-mem_BCFtools** | 2,993,361 | 14 | 2,340,967 | 4,128,734 | 253,163 | 17 | 168,957 | 351,611 |
| **BWA-mem_FreeBayes** | 1,367,505 | 8 | 1,146,060 | 1,682,846 | 187,123 | 16 | 128,939 | 253,860 |
| **BWA-mem_VarScan** | 292,727 | 18 | 183,726 | 413,002 | 203,583 | 20 | 122,654 | 287,458 |
| **GSNAP_BCFtools** | 5,079,109 | 17 | 3,834,097 | 7,135,397 | 275,246 | 18 | 164,161 | 384,081 |
| **GSNAP_FreeBayes** | 2,052,114 | 9 | 1,724,375 | 2,517,808 | 291,472 | 16 | 195,253 | 395,371 |
| **GSNAP_VarScan** | 247,817 | 19 | 142,305 | 353,044 | 173,411 | 20 | 94,414 | 246,536 |
| **Hisat2_BCFtools** | 2,386,939 | 17 | 1,825,369 | 3,393,135 | 120,409 | 16 | 78,865 | 161,888 |
| **Hisat2_FreeBayes** | 1,239,130 | 8 | 975,311 | 1,458,064 | 177,955 | 16 | 118,546 | 234,396 |
| **Hisat2_VarScan** | 143,629 | 17 | 88,545 | 195,879 | 98,729 | 19 | 57,157 | 137,862 |
| **Novoalign_BCFtools** | 3,094,586 | 14 | 2,412,166 | 4,254,600 | 211,377 | 18 | 139,225 | 298,939 |
| **Novoalign_FreeBayes** | 1,259,015 | 8 | 1,063,957 | 1,481,847 | 182,645 | 17 | 122,994 | 247,641 |
| **Novoalign_VarScan** | 219,349 | 19 | 136,191 | 307,587 | 153,475 | 20 | 90,504 | 215,220 |
| **STAR_BCFtools** | 733,697 | 10 | 601,905 | 960,377 | 172,776 | 17 | 112,219 | 233,877 |
| **STAR_FreeBayes** | 1,581,001 | 8 | 1,324,930 | 1,915,956 | 222,865 | 16 | 161,444 | 296,046 |
| **STAR_VarScan** | 278,141 | 18 | 178,871 | 390,579 | 193,908 | 19 | 120,087 | 271,130 |
