# Supplementary material for: Multiple Variant Calling Pipelines in Wheat Whole Exome Sequencing
Source: Int J Mol Sci. 2021 Sep 27;22(19):10400. doi: 10.3390/ijms221910400 (PMC8509018; doi:10.3390/ijms221910400)
Supplement: Supplementary file 1 [file ijms-22-10400-s001.zip › Table_S3.pdf]

Table S3. Accuracy metrics of the performance of variant calling pipelines at the sample and population level. Optimum pipelines providing above the average scores were colored red. The maximum and minimum values for each accuracy metrics were bolded. TP: True Positive, PRE: precision, SN: sensitivity, F1: F1 score.

	per Sample				Population			
	**TP**	**PRE**	**SN**	**F1**	**TP**	**PRE**	**SN**	**F1**
Bowtie2_BCFtools	91,324	0.689	0.019	0.036	480,701	0.579	0.091	0.157
**Bowtie2_FreeBayes**	25,580	**0.729**	0.005	**0.011**	128,948	**0.611**	0.026	0.050
Bowtie2_VarScan	92,902	0.481	0.019	0.036	512,540	0.313	0.096	0.147
Bowtie2-local_BCFtools	98,712	0.633	0.020	0.039	533,233	0.505	0.100	0.167
**Bowtie2-local_FreeBayes**	**17,524**	0.625	**0.004**	0.007	**93,499**	0.517	**0.019**	**0.037**
Bowtie2-local_VarScan	97,988	0.469	0.020	0.038	532,996	0.333	0.100	0.154
BWA_BCFtools	103,502	0.555	0.021	0.041	543,147	0.428	0.101	0.164
BWA_FreeBayes	65,959	0.597	0.014	0.026	355,538	0.401	0.069	0.118
BWA_VarScan	88,453	0.547	0.018	0.035	480,080	0.414	0.091	0.149
**BWA-mem_BCFtools**	**118,265**	0.469	**0.024**	**0.046**	**613,023**	0.331	**0.113**	**0.169**
BWA-mem_FreeBayes	91,479	0.490	0.019	0.036	477,976	0.319	0.090	0.141
BWA-mem_VarScan	98,567	0.486	0.020	0.038	530,111	0.347	0.099	0.154
GSNAP_BCFtools	107,157	0.391	0.022	0.041	603,703	0.248	0.112	0.154
**GSNAP_FreeBayes**	98,190	**0.337**	0.020	0.038	558,911	**0.201**	0.104	0.137
GSNAP_VarScan	88,274	0.510	0.018	0.035	492,702	0.371	0.093	0.149
Hisat2_BCFtools	69,191	0.575	0.014	0.028	378,768	0.427	0.073	0.125
Hisat2_FreeBayes	77,726	0.437	0.016	0.031	431,219	0.271	0.082	0.126
Hisat2_VarScan	62,516	0.634	0.013	0.025	344,120	0.507	0.067	0.118
NovoAlign_BCFtools	96,848	0.459	0.020	0.038	529,660	0.327	0.099	0.152
NovoAlign_FreeBayes	83,969	0.460	0.017	0.033	456,459	0.305	0.087	0.135
NovoAlign_VarScan	81,855	0.535	0.017	0.032	458,081	0.411	0.087	0.144
STAR_BCFtools	101,310	0.588	0.021	0.040	545,305	0.439	0.102	0.165
STAR_FreeBayes	93,115	0.418	0.019	0.036	508,848	0.253	0.096	0.139
STAR_VarScan	98,747	0.511	0.020	0.039	535,122	0.373	0.100	0.158
